# Disitamab Vedotin (RC48) combined with bevacizumab for treatment of HR-negative/HER2-positive metastatic breast cancer with liver and brain involvement: A case report

**DOI:** 10.3389/fonc.2023.1245701

**Published:** 2023-08-28

**Authors:** Fei Qu, Qian Liu, Rongrong Lu, Wei Li

**Affiliations:** ^1^ Department of Oncology, The First Affiliated Hospital of Nanjing Medical University, Nanjing, China; ^2^ The First Clinical College of Nanjing Medical University, Nanjing, China

**Keywords:** HER2-positive, brain metastatic breast cancer, anti-angiogenesis, RC48, case report

## Abstract

**Background:**

The overexpression of human epidermal growth factor receptor 2 (HER2) is strongly correlated with an elevated risk of developing distant metastases, particularly brain metastases, in breast cancer (BC) cases. RC48 (also known as Disitamab vedotin), represents a promising antibody-drug conjugate (ADC), that comprises three well-defined components: hertuzumab against the prominent tumor target-HER2, monomethyl auristatin E (MMAE) and a cleavable linker. Preclinical studies have demonstrated its robust antitumor activity in BC patient-derived xenograft models with HER2-positive or HER2-low expression. Additionally, antiangiogenic drugs like bevacizumab have shown potential efficacy on advanced BC *via* inhibiting pathological neovascularizationits.

**Case presentation:**

Here, we will share our experience in treating a 49-year-old woman initially diagnosed with stage IV breast cancer characterized by hormone receptor (HR)-negativity and HER2-positivity. This complex case entailed brain and liver metastases, and the patient exhibited resistance to various HER2-targeted treatment regimens. Finally, the patient received RC48 plus bevacizumab as the advanced forth-line treatment, which was well tolerated with no observed toxicities. Subsequent radiological assessments revealed remarkable regression in the brain metastatic lesions, classified as having partial response based on the RECIST 1.1 system. The period of progression-free survival (PFS) was 7 months.

**Conclusion:**

The present study underscores the efficacy of systemic treatment with RC48 in conjunction, showcasing substantial enhancement in both radiographic indicators and clinical symptomatology among patients with brain metastatic breast cancer (BMBC). More specifically, the sequential application of ADCs in combination with antiangiogenics presents a novel avenue for advancing the treatment landscape of metastatic BC.

## Introduction

Breast cancer is now ranked first in terms of incidence and second as the leading cause of mortality among women worldwide. Within this context, HER2-positive BC comprises approximately 25% of all subtypes (1). These tumors express high levels of HER2, which is recognized as a cancer-driving gene and an independent prognostic determinant for BC (2). The emergence of brain metastases (BMs) presents a major clinical challenge that tends to develop in up to approximately 40% of individuals with HER2-positive status. To date, the treatment options for BMBC patients remain limited, focusing on local treatment, including whole-brain radiotherapy (WBRT) and, if applicable, surgical removal of the intracranial metastatic lesions (3). While initially effective, these interventions are linked to significant neurological or systemic repercussions attributed to the metastatic and infiltrative growth patterns, subsequently undermining the overall quality of life and offering limited scope for substantial prognosis enhancement (4). Hence, additional treatment options, including systemic therapy, are warranted to prolong survival. Several retrospective research findings have shown that patients with BMs who received HER2-targeted therapy experienced a prolonged median survival period compared to those who did not receive HER2-targeted therapy (5). The concurrent administration of docetaxel alongside trastuzumab and pertuzumab has emerged as a recommended standard front-line treatment option for patients with HER2-positive metastatic breast cancer (mBC) (6). However, the efficacy of this regimen within the context of BM patients is significantly limited due to the prevailing notion that monoclonal antibodies, as biomolecules, encounter difficulties in traversing the blood-brain barrier (BBB). In recent times, the field of clinical practice has witnessed the introduction of ado-trastuzumab (T-DM1), trastuzumab deruxtecan (T-DXd, DS8201), and a regimen based on the tyrosine kinase inhibitor pyrotinib. These have demonstrated encouraging results as second-line therapies for patients with untreated BMs. However, third-line therapy in HER2-positive BMBC patients has been met with controversies (7), and the identification of new therapeutic strategies is urgently required.

In the early 1900s, Paul Ehrlich conceptualized the notion of a “magic bullet”. Against this historical backdrop, a novel category of medications for solid and hematologic malignancies emerged: namely, antibody-drug conjugates (ADCs) (8). Following drug administration, the ADC formulation contains three predominant circulating components: the conjugate (which constitutes the majority of the composition), specific antibodies, and free payload molecules. ADCs are created with the purpose of directly accomplishing their desired cell-structural objectives while being non-aggressive to healthy tissues, thereby mitigating systemic toxicities. Disitamab Vedotin (RC48), as a Chinese original HER2-targeting ADC, developed by Rongchang Biology, has shown promising potential in HER2‐overexpressing locally advanced or metastatic gastric cancer and uroepithelial cancer, based on the results of RC48 C008, C005 and C009 studies (9). RC48 not only destroys cancer cells accurately and directly but also has the capacity to exert a “bystander effect” on neighboring cells (10, 11). This characteristic of internalized ADCs necessitates the diffusion of lipophilic payloads through cell membranes thereby playing a pivotal role in the efficacy of RC48 against malignancies characterized by heterogeneous target antigen expression. The pooled results of two studies (C001 and C003 CANCER) presented in the 2021 American Society of Clinical Oncology (ASCO) Meeting, provide compelling insights. Among 118 mBC patients treated with RC48, 70 cases were HER2-positive and 48 cases had low expression of HER2. Notably, 47 patients had previously undergone treatment with three or more chemotherapy regimens (12). The use of RC48 monotherapy exhibited discernible indicators of therapeutic efficacy, resulting in complete or partial remission for nearly 40% of patients undergoing treatment in later-line stages. In June 2021, the Center for Drug Evaluation in China granted a new indication for RC48 as a prospective breakthrough therapy for HER2-positive mBC patients with liver metastases who had been previously treated with trastuzumab and paclitaxel.

In addition, tumors require a constant and abundant supply of blood to sustain cellular replication and energy metabolism. Vascular endothelial growth factor (VEGF) is critical in modulating angiogenesis both in physiologic and pathologic conditions. Excessive VEGF signaling can induce abnormal angiogenesis within tumor tissues, due to increased tumor vessel dilatation, permeability, and leakage (12). Substantial support from laboratory and clinical investigations has highlighted the inverse relationship between VEGF expression levels and the clinical outcomes of breast cancer (BC) patients. Moreover, in preclinical models, brain metastases from BC were found to display higher microvessel density compared to their corresponding primary tumors (13), supporting the hypothesis that brain metastases may be more dependent on angiogenesis than extracranial breast tumors. A study of the possible influence of T-DM1 on blood vessels of HER2-amplified breast cancer brain metastasis model revealed a trend towards reduced vessel diameter and vascular fraction, thus preventing the formation of brain metastases and delaying their growth rate (14). In HER2-positive BMBC patients, the interplay between HER2 and VEGF pathways has laid a theoretical foundation for potentially effective therapies combining antiangiogenic drugs with HER2-targeting therapies (15, 16). The novel antiangiogenic drug bevacizumab, which is an intravenously administered recombinant monoclonal antibody was developed with VEGF in mind (17). In clinical practice, bevacizumab has exhibited impressive efficacy alongside manageable safety profiles in heavily treated patients with metastatic breast cancer (18).

However, to our knowledge, there are no published reports documenting the application of RC48 combined with antiangiogenic drugs for BC treatment. This report will present visible tumor shrinkage and substantial clinical improvement in responding to RC48 combined with bevacizumab treatment in one BC patient with brain and liver metastases.

## Case presentation

**Figure 1:**
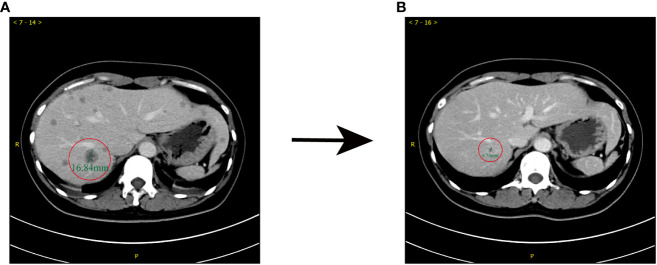
Comparison of enhanced CT prior to (June, 2019) and after (December, 2019) first-line treatment. **(A)** Before the administration of TCbH, abnormal small nodular signals were observed in the right lobe of the liver, which was 16 mm in diameter. **(B)** After 5 cycles of TCbH, shrinkage of the liver metastasis was observed; the diameter of the lesion was 6 mm.

In June 2019, a 49-year-old female patient presented at Jiangsu Province Hospital, Nanjing Medical University (Nanjing, China), with complaints of fatigue and thickening of the skin on her breasts. The patient reported no discomfort such as obvious local aching or distending pain during the menstrual phase. Upon physical examination, a palpable, firm, and irregular mass was detected in the right breast, accompanied by skin erythema. Ultrasound examination of the breast identified a 4.7×4.6×2.7 cm^3^ mass in the right breast, classified as level 6 according to the breast imaging reporting and data system (BI-RADS). Additionally, enlarged lymph nodes were observed in regions I, II, and III on the right axilla, suggesting a high probability of metastasis. Pathological analysis of the core needle biopsy specimen extracted from the mass revealed non-specific invasive breast cancer. Immunohistochemistry (IHC; HercepTest, Dako, Denmark) confirmed estrogen receptor (ER) -negative/progesterone receptor (PR) -negative and HER2 (3+) disease with Ki-67 of 25%+. A computed tomography (CT) scan revealed multiple metastases in the liver (**Figure 1A**), while a whole-body bone scintigraphy using 99m Tc-MDP did not reveal any notable abnormalities. The patient had no family history of breast or ovarian cancer, no medical history of radiation or hormonal replacement therapy, and no history of smoking, drinking, or other harmful behaviors. Ultimately, the patient was diagnosed with grade 3 invasive breast cancer, accompanied by right axillary lymph node and liver metastasis, cT2N2M1, stage IV.

Starting from July 6, 2019, until March 10, 2020, the patient underwent advanced first-line treatment with the TCbH regimen, a combination of nab−docetaxel, carboplatin, and trastuzumab. Efficacy evaluation was a partial response (PR) at that time. However, by February 2020, the patient began to experience lower limb muscle strength dysfunction, dizziness, and impaired fine motor coordination skills in both hands. Subsequently, a contrast−enhanced brain magnetic resonance imaging (MRI) revealed multiple brain metastases situated in the right parietal lobe and the left fronto-temporo-parieto-occipital lobe. The lesions were accompanied by obvious edema and the midline structure shifted to the right; the left lateral ventricle moved to the right under pressure. Despite substantial primary tumor and brain metastases growth and extensive tumor burden, the metastatic lesions in the liver gradually shrank and disappeared (**Figure 1B**). The final assessment of therapeutic efficacy resulted in the identification of progressive disease (PD). After a multi-disciplinary discussion, consideration was given to the following points: i) Radiographic examination revealed approximately five huge brain metastases. These metastases were surrounded by marked edema with a high risk of raised intracranial pressure; ii) Two ulcerations were detected in tumors in the upper-outer quadrant of the breast, accompanied by persistent malodorous surface discharge. The Breast Surgery department indicated that it might be able to perform palliative surgery, but the patient’s condition should be fully informed; iii) systemic chemotherapy was necessary.

After careful consideration, the patient and the patient’s family accepted our comprehensive treatment regimens. The patient accepted the standard second-line chemotherapy combining pyrotinib and capecitabine, starting on March 22, 2020. Concurrently, the patient received WBRT (54 Gy total, administered in 20 fractions, five times a week) and was administered with mannitol and dexamethasone to reduce intracranial pressure. On June 19, 2020, a modified radical mastectomy was performed and sent for histopathological examination. The postoperative TNM classification was ypT2N1aM1 G3 R0. The tumor was ER (-), PR (-), Her-2 (2+) and Ki67 (40%+). FISH analysis confirmed HER2 amplification. Notably, a BRCA test was not conducted. Postoperatively, the patient continued to be treated with the “pyrotinib + capecitabine” regimen. However, CA-153 levels elevated continuously and an MRI reexamination indicated enlarged and increased brain metastases on February 23, 2021, again indicating PD. Systemic CT suggested that liver metastases remained stable.

**Figure 2:**
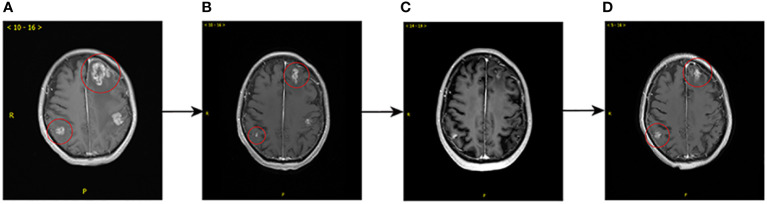
Comparison of brain MRI prior to (February, 2022) and after (September, 2022) forth-line treatment. **(A)** Before the administration RC48 plus bevacizumab, multiple brain metastases in the bilateral cerebellar hemispheres, the right parietal lobe and the left fronto-temporo-parieto-occipital lobe were observed, with obviously annular and nodular enhancement, the larger of which measured 2.6 x 2.5 cm2. **(B)** After 4 cycles, shrinkage of the brain metastases was observed, the larger of which measured 1.5 x 1.3 cm2, with diminished enhancement of some lesions. **(C)** After 7 cycles, further shrinkage of the brain metastases was observed. **(D)** After 13 cycles, MRI showed the volume of some brain metastatic sites increased with marked annular and nodular enhancement, the larger of which measured 1.7 x 1.3 cm2, which suggested local progression of tumor.

T-DXd, as the advanced third-line treatment was applied from March 2021. The initial dose of T-DXd was 200mg, administered every three weeks. In addition, the patient underwent gamma knife treatment for cerebellar metastasis on September 24, 2021. Enhanced CT and MRI with dynamic review during chemotherapy revealed a reduction in the size of intracranial and extracranial lesions. Overall, the best follow-up evaluations, conducted every two months, suggested a stable disease (SD) and the PFS was 11.5 months for the third-line treatment. In February 2022, MRI showed that the brain metastases were enlarged (**Figure 2A**), representing tumor progression. RC48 was begun after obtaining informed consent. RC48 (116mg, q2w) combined with bevacizumab (290 mg, q2w) as the advanced forth-line treatment, was administered. Notably, within two months, a reduction in size was observed on MRI scans, transitioning from 2.6 x 2.5 cm² to 1.5 x 1.3 cm² within the right parietal lobe (**Figure 2B**). As of June 28, 2022, the MRI demonstrated a further decrease in the dimensions of residual lesions (**Figure 2C**). At the time of writing, due to the re-progression of brain metastases (**Figure 2D**), the patient was treated with RC48 combined with bevacizumab and pyrotinib. This marked the start of the fifth-line treatment, from September 2022.

Furthermore, we systematically reviewed routine blood tests, tumor markers, biochemical indexes, as well as liver and kidney function electrolyte indicators during the administration of RC48 and bevacizumab, all of which were within the normal range, although they were changeset at different stages of the treatment. Apart from minor liver injury and nausea, which were controlled by symptomatic treatment, no other obvious drug-related toxicity was reported during the treatment. The therapeutic effect was that of partial remission. The patient received a total of 13 cycles of RC48 combined with bevacizumab and had a PFS (from treatment initiation to progression of BM) of 7 months. The summary of the patient’s post-RC48 plus bevacizumab treatment results for key indicators is provided in **Table 1**, while **Table 2** outlines the patient’s treatment timeline. Notably, the patient has provided her consent for the publication of this case report.

**Table 1:** Laboratory data of the patient after RC48 plus bevacizumab administration (September 21, 2022).

Hematology		Normal range	Biochemistry		Normal range
White blood cell	4.64×10^9/L	3.50–9.50	Total protein	63.3↓g/L	65.0–85.0
Neutrophil	41.10%	40.00–75.00	Albumin	38.6↓g/L	40.0–55.0
Eosinophil	2.60%	0.40–8.00	Total bilirubin	11.2μmol/L	5.1–19.0
Basophil	2.20↑%	0.00–1.00	AST	26.9U/L	13.0–35.0
Monophil	8.10%	3.00–10.00	ALT	38.7U/L	7.0–40.0
Lymphocyte	36.20%	20.00–50.00	LDH	194U/L	140–271
Red blood cell	3.84x10^12/L	3.80–5.10	UA	255μmol/L	155–357
Hemoglobin	112↓g/L	115–150	Creatinine	48.2μmol/L	44.0–133.0
Platelet	346x10^9/L	125–350	Na	142.5mmol/L	137.0-147.0
Hematology		Normal range	K	3.56mmol/L	3.50-5.30
CEA	7.16↑ng/ml	<4.7	Ca	2.21mmol/L	2.20-2.65
CA15-3	20.90U/ml	<25.0			
CA125	7.8U/ml	<35.00			

**Table 2:** Treatment history of the patient.

Line	1st	2nd	3rd	4th
Regimen	TCbH	Pyrotinib + Capecitabine	T-DXd	RC48+bevacizumab
Treatment period (months)	9	11	11	7
Best of response	PR	PR	SD	PR

## Discussion

Brain metastases are a prevalent form of malignant intracranial tumors in adults, often leading to severe complications for individuals with solid cancers, ultimately impacting their survival prospects and quality of life. While trastuzumab-based therapy has demonstrated noteworthy advancements in the survival rates of patients with HER2-positive breast cancer subtypes, its role in preventing disease recurrence or progression into the brain remains limited (19). This phenomenon has been coined the “HER2 paradigm,” wherein patients who exhibit exceptional systemic disease control or the absence of extracranial disease paradoxically experience an increase in the incidence of brain metastases (20). A possible reason for the “HER2 paradigm” is that some novel compounds including trastuzumab do not, or only partially, penetrate the BBB, effectively. The potential for the brain to serve as a “sanctuary site” for cancer arises from the possibility that therapeutic agents may not effectively target tumor cells that have successfully infiltrated the brain. Therefore, the treatment of HER2-targeting drug-resistant patients is an important clinical challenge.

Studies on antibody-drug conjugates (ADCs) targeting HER2, such as T-DM1 and T-DXd have reported promising outcomes. An exploratory analysis of central nervous system metastasis in the KAMILLA study, a prospective, single-arm phase IIIb clinical trial (21), shows that T-DM1 may prolong PFS and OS among patients with BMBC and is well-tolerated in this population. Moreover, T-DXd is characterized by a novel enzyme-cleavable linker and the payload (DXd) with high membrane permeability, thus emerging as a promising agent for the treatment of HER2+ mBC, supported by a series of clinical trials. The DESTINY-Breast 03 study, which included a subgroup of patients with stable brain metastases at baseline, revealed indicated that T-DXd achieved encouraging therapeutic outcomes for BMBC patients, with an mPFS of 15 months (22). TUXEDO-1, a prospective, single-arm, phase II clinical trial, verified the efficacy of T-DXd in HER2+ BCBM patients who were untreated or had progressed after prior local treatment (23). Among 15 eligible patients, the intracranial response rate (RR) was 73.3% (11/15) and the clinical benefit rate (CBR) was 92.9% (13/14).

The impact of BBB breakdown on the efficacy of systemic therapies against brain metastases remains a topic of debate. However, recent findings suggest that BBB disruption occurs during the progression of brain metastases, thereby facilitating the enhanced penetration of numerous drugs. Radiolabeling studies of ADCs showed drug aggregation in HER2-positive BMBC, suggesting that the compromised BBB may allow for the passage of ADCs (24). Furthermore, the pathological effects of WBRT on the BBB may increase the permeability of the drugs to exert therapeutic effects. As described by Stemmler et al., elevated levels of trastuzumab in cerebrospinal fluid (CSF) in the presence of impaired BBB following WBRT. Specifically, the serum/CSF trastuzumab ratio before and after WBRT was 420: 1 and 76:1 under conditions of impairment of the BBB, respectively (25). These data theoretically indicate that patients with HER2-positive BMBC can benefit from subsequent anti-HER2 therapy based on WBRT.

In addition, angiogenesis is closely related to HER2 signal transduction at the molecular level. In a preclinical study, the authors observed that VEGFR2-positive stromal vessel counts were higher in HER2-positive BC compared to other subtypes (26). In other words, the influence of HER2 on oncogenesis and metastasis may be partially mediated by the stimulation of angiogenesis, providing a solid theoretical basis for the use of antiangiogenic drugs in HER2-positive BC. Preclinical cancer models have suggested that antiangiogenic agents may facilitate ADC penetration and exposure of tumor cells by starving cancer cells and normalizing the aberrant structure and function of tumor vasculature (27). The blend of anetumab ravtansine or mirvetuximab soravtansine with bevacizumab has yielded potent effects and complete responses in preclinical ovarian cancer models (28, 29). These preclinical findings were recapitulated by a Phase 1b trail evaluating the clinical activity and safety of mirvetuximab soravtansine in combination with bevacizumab in heavily pretreated, platinum-resistant, FRα-positive, ovarian cancer patients, with a confirmed ORR of 39% and a median PFS of 6.9 months, which was superior to the benchmark values of the pivotal AURELIA trial (27%). The combination was more favorable in patients who were bevacizumab-naïve, less heavily pretreated (1-2 prior lines), and whose tumors exhibited medium/high FRα expression (ORR, 56%; PFS, 9.9 months). Reportably, only 4.5% of patients were observed to have grade 3 thrombocytopenia and 1.5% to have neutropenia, the anticipated overlapping toxicities (30).

In this context, we present a case in which the combination of RC48 with bevacizumab showed effectiveness in one patient with HER2-positive BC and effectively controlled refractory brain metastases after the failure of pyrotinib treatment. In addition, the patient also received WBRT and gamma knife radiotherapy for brain metastases before RC48 treatment. Surprisingly, this combination regimen produced remarkable results. Brain MRI indicated that the reduction of the brain metastases from breast cancer and alleviation of the brain edema lasted 7 months. The patient’s headache and vomiting symptoms improved significantly. This may be due to the synergistic antitumor effect of ADC and antiangiogenic drugs through the BBB.

Moreover, as more and more ADCs are developed and enter clinical settings, some of which target the same antigen or have comparable payloads, determining the optimal therapeutic sequencing of these agents represents an upcoming challenge. Sequential ADC administration may be practical and efficient. In the present case, RC48 has demonstrated activity in the patient who had previously received T-DXd, most likely due to the different mechanisms underpinning the payloads of these two ADCs. Other reasons for the phenomenon are unclear and warrant further exploration.

Therefore, based on the above analysis, there is reason to believe that administering RC48 with bevacizumab is feasible and effective. Combined therapy can enhance the activity of a single drug, reduce the risk of drug resistance and further improve its efficacy (31). Moreover, ongoing efforts encompass an array of combinatorial strategies, extensively explored through both preclinical models and clinical investigations, including coadministration with immune checkpoint inhibitors (ICIs), tyrosine kinase inhibitors (TKIs), large or small molecule anti-angiogenic drugs, DNA-damage response agents, and metronomic chemotherapy, thus integrating and optimizing existing clinical treatment options.

## Conclusion

In recent times, there has been a notable surge of interest among clinicians in addressing the challenges posed by patients grappling with HR-negative/HER2-positive brain metastatic breast cancer (BMBC), particularly owing to the involvement of multiple organs and poor physical condition. The application of RC48 is anticipated to exert a substantial and meaningful influence on the prognosis of these individuals. Additional translational and clinical investigations are imperative to ascertain whether RC48’s impact on BMBC will culminate in enduring advantages for disease management. Furthermore, it is crucial to identify optimal partners that offer additive or synergistic benefits, devoid of overlapping toxicities when administered alongside ADCs.

## Data availability statement

The original contributions presented in the study are included in the article/supplementary material. Further inquiries can be directed to the corresponding author.

## Ethics statement

Written informed consent was obtained from the individual(s) for the publication of any potentially identifiable images or data included in this article.

## Author contributions

FQ and QL analyzed the cases and radiological images. FQ and RL participated in writing. WL reviewed the manuscript. All authors contributed to the article and approved the submitted version.
